# Supplementary material for: Identification of Polyunsaturated Fatty Acids Synthesis Pathways in the Toxic Dinophyte Alexandrium minutum Using 13C-Labelling
Source: Biomolecules. 2020 Oct 8;10(10):1428. doi: 10.3390/biom10101428 (PMC7600785; doi:10.3390/biom10101428)
Supplement: Supplementary file 1 [file biomolecules-10-01428-s001.zip › biomolecules-930980 Re 1 suppl/Remize et al_Supplementary Data.pdf]

SUPPLEMENTARY DATA - GC-FID CHROMATOGRAMS

SUPELCO 37 – DB-5 (05.06.2018) [A] scale 80000 µV [B] scale 20000 µV

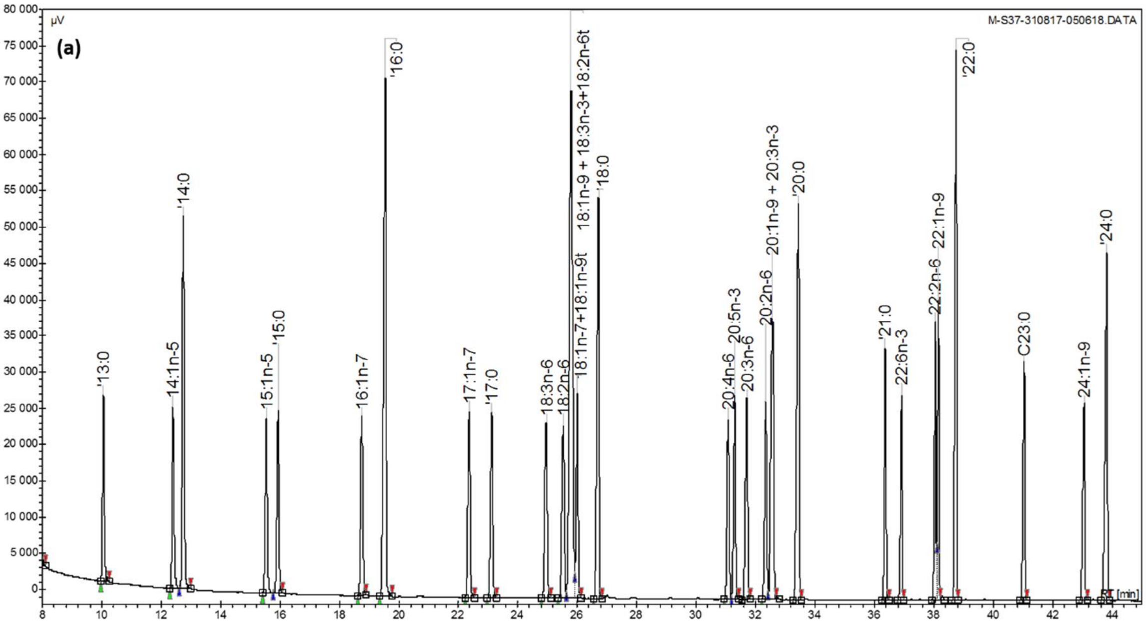

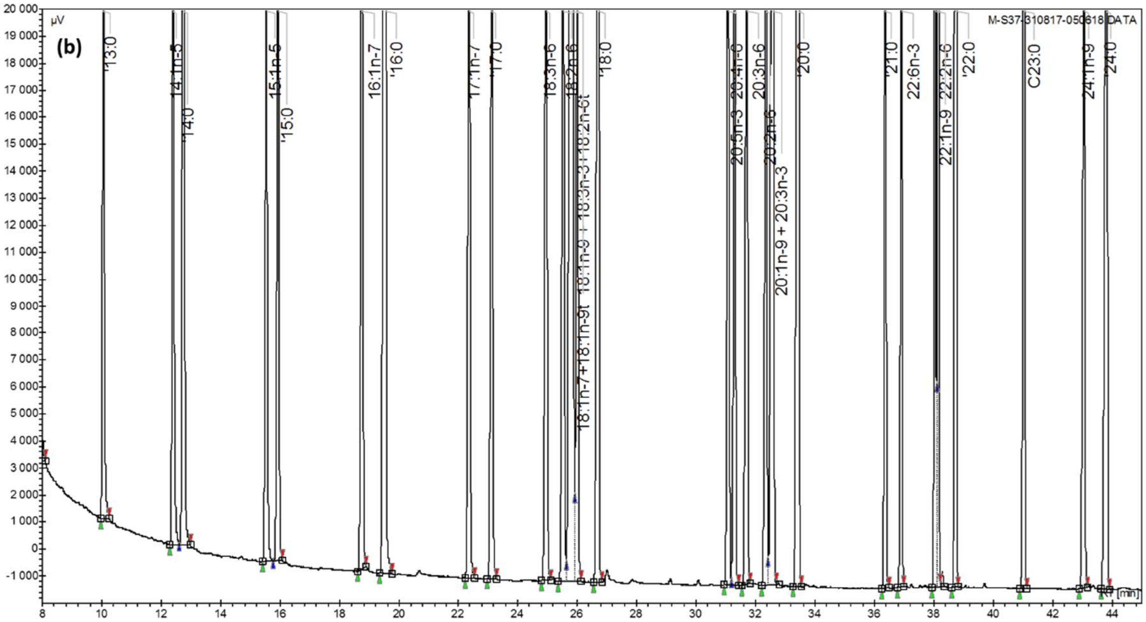

SUPELCO 37 – DB-5 (03.08.2018) [A] scale 80000 µV [B] scale 20000 µV

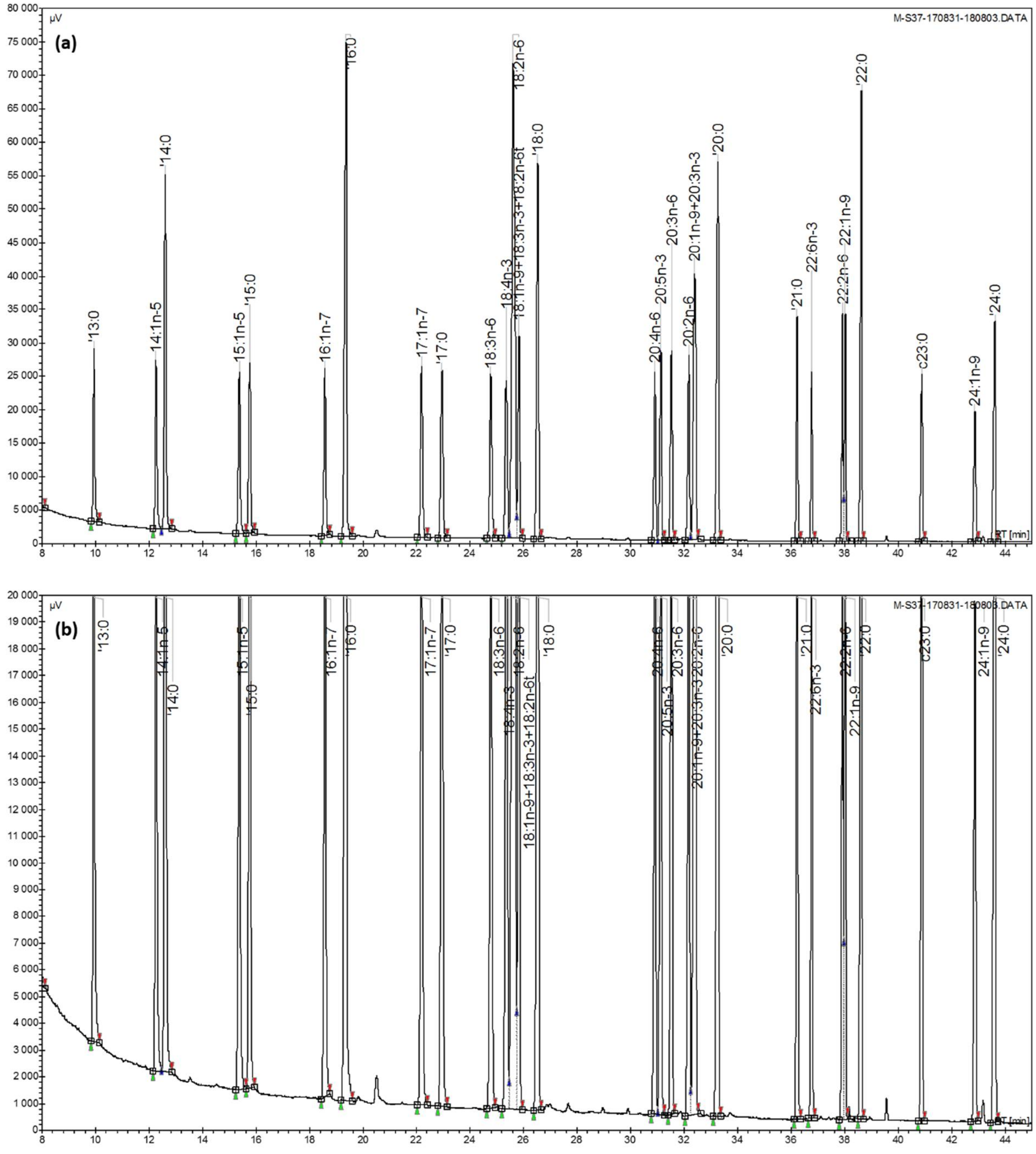

PUFAs No.1 – DB-5 (05.06.2018) [A] scale 110000 µV [B] scale 20000 µV

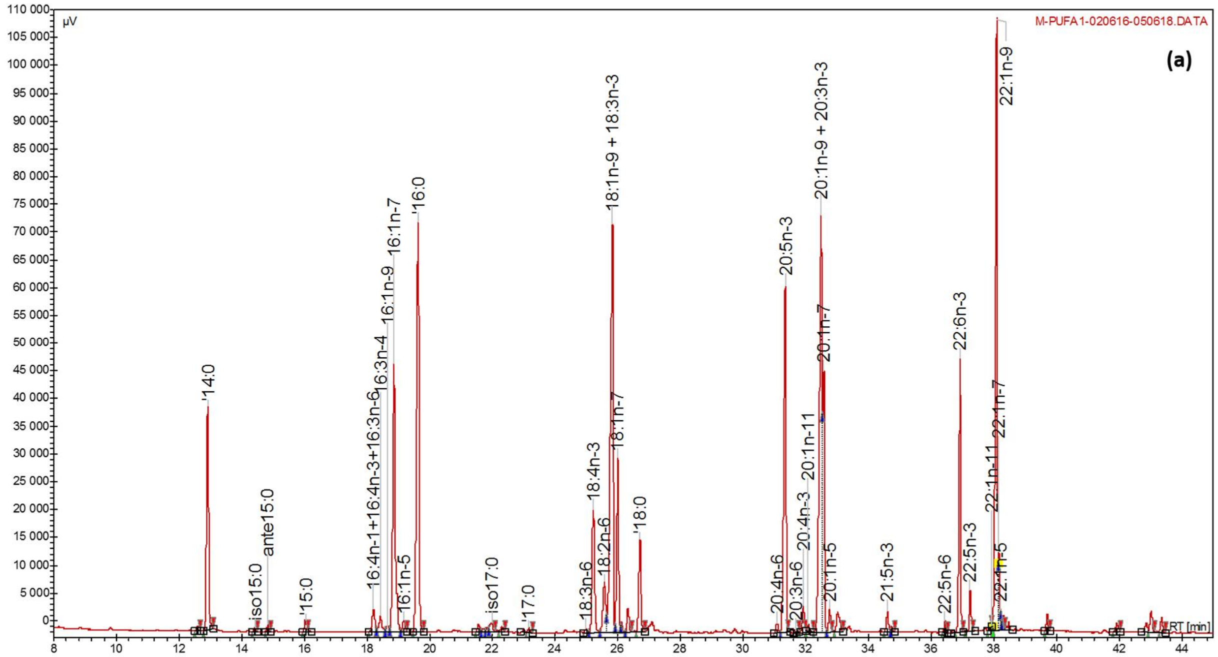

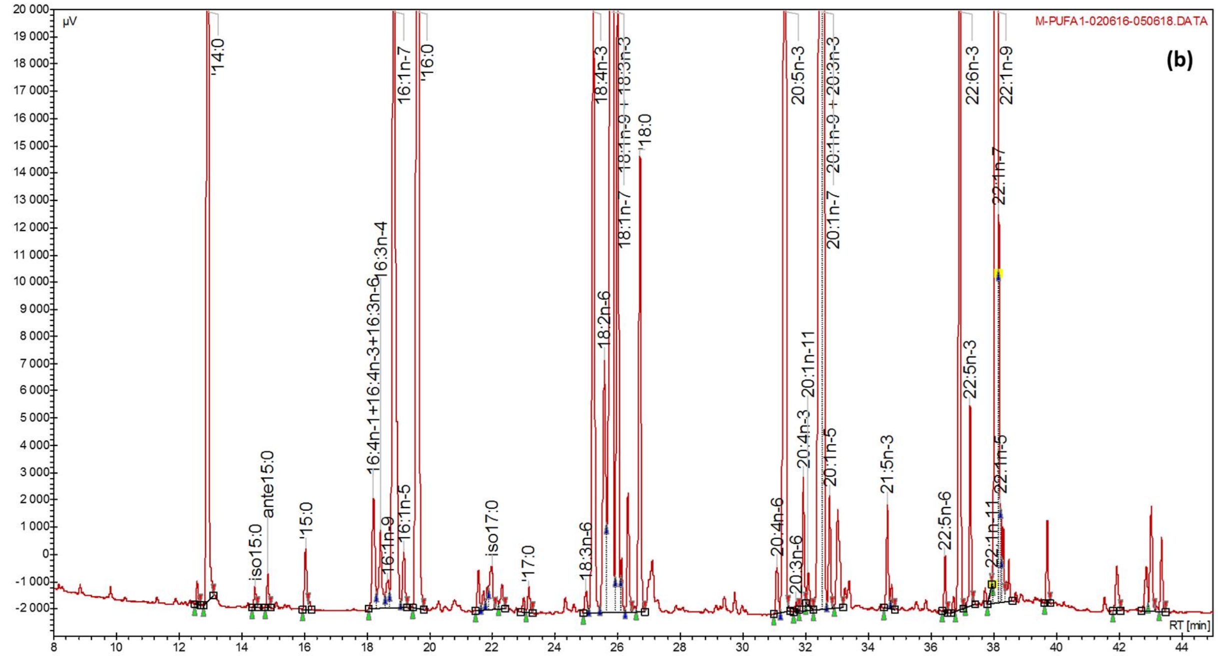

PUFAs No.1 – DB-5 (03.08.2018) [A] scale 100000 µV [B] scale 20000 µV

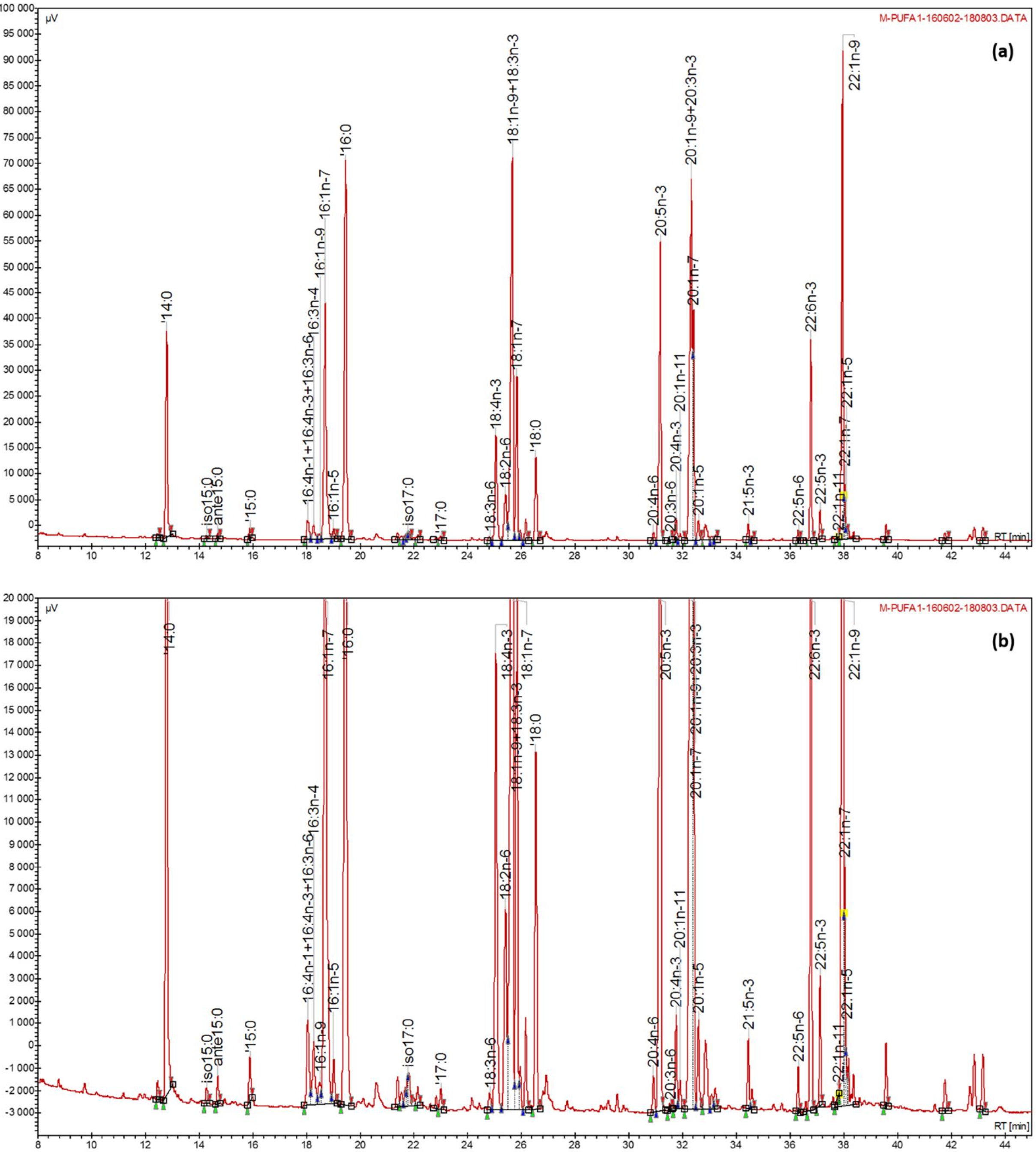

PUFAs No.3 – DB-5 (05.06.2018) [A] scale 110000 µV [B] scale 20000 µV

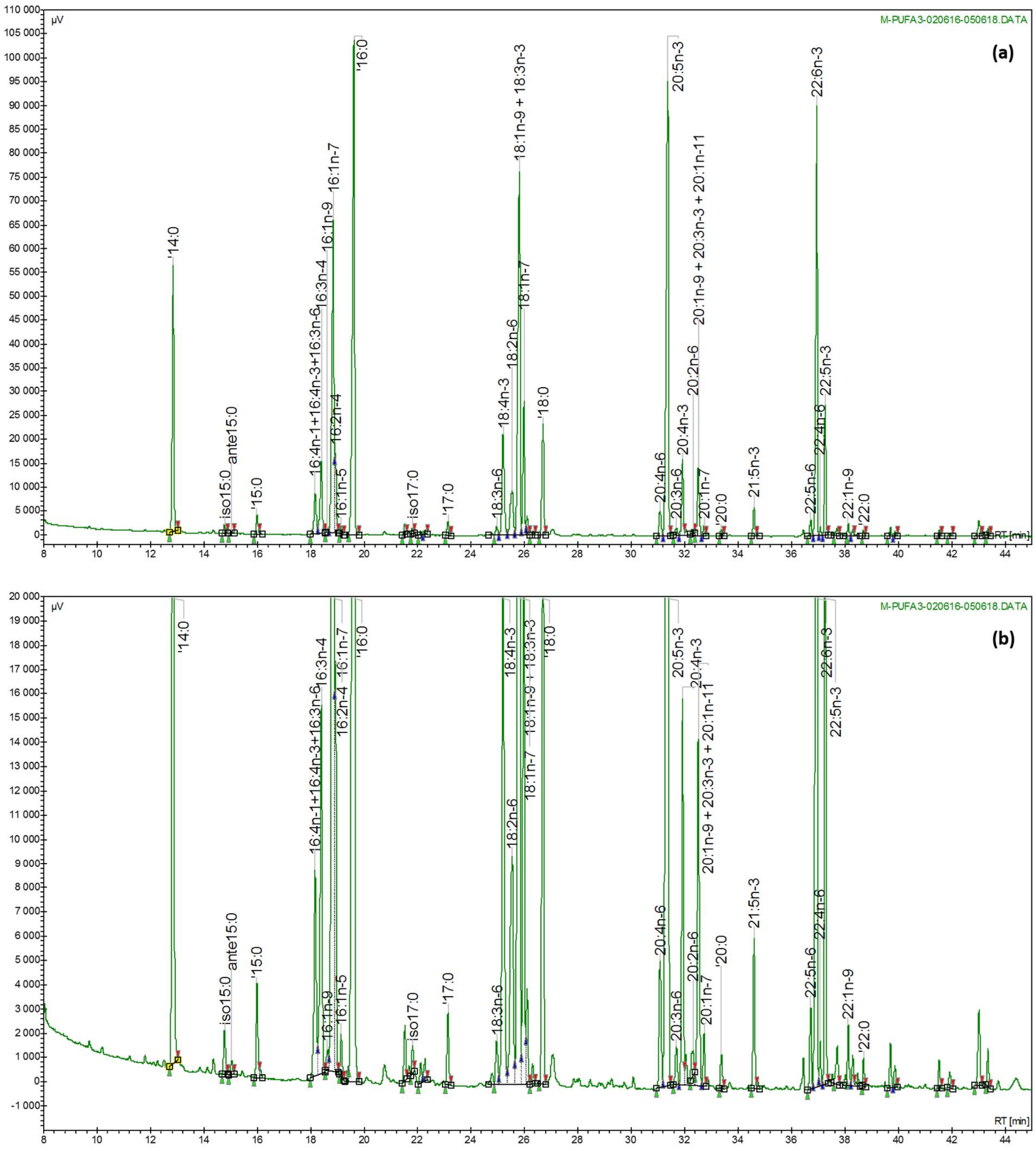

PUFAs No.3 – DB-5 (03.08.2018) [A] scale 110000 μV [B] scale 20000 μV

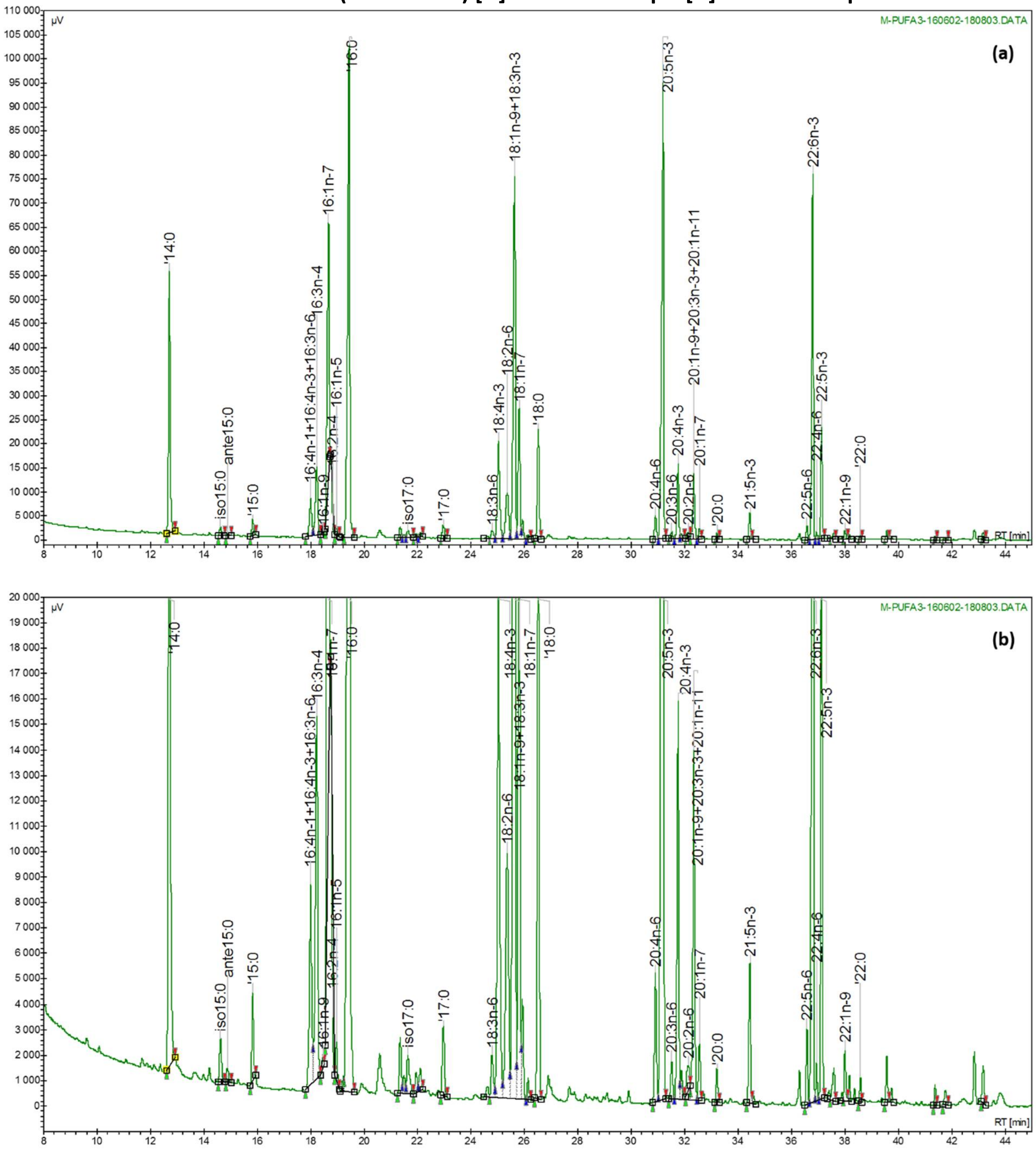

SAMPLE *Alexandrium minutum* Neutral Lipid (NL) – DB-5 scale 14000 µV

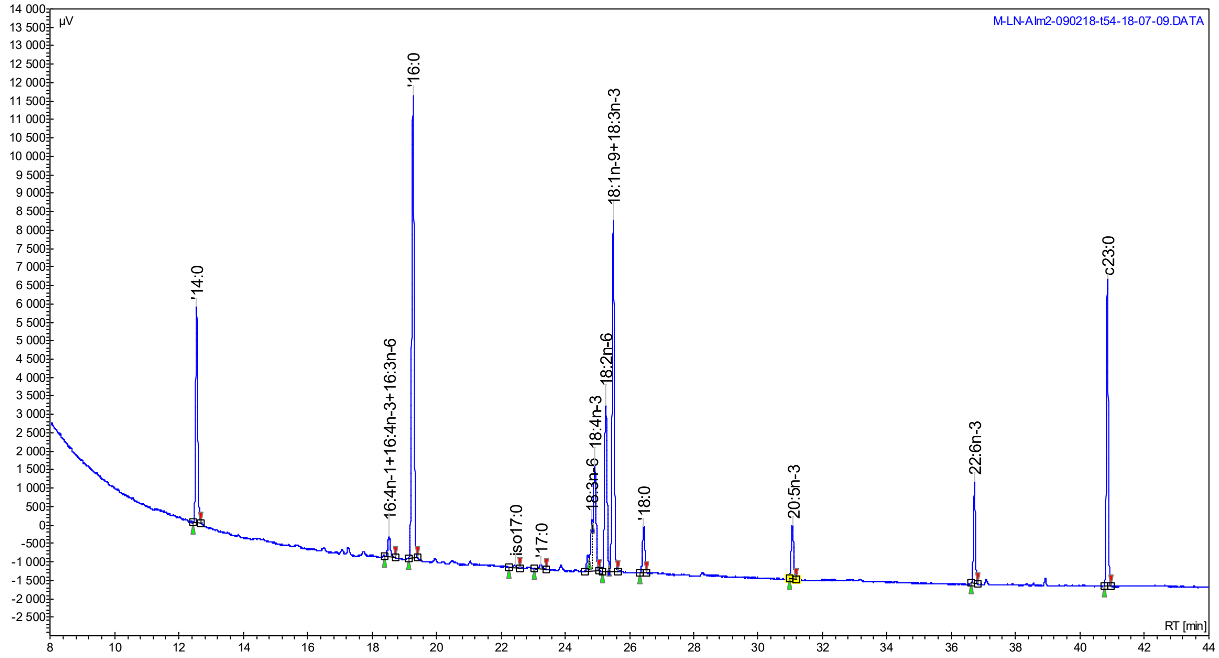

SAMPLE *Alexandrium minutum* Polar Lipid (PL) – DB-5 scale 30000 µV

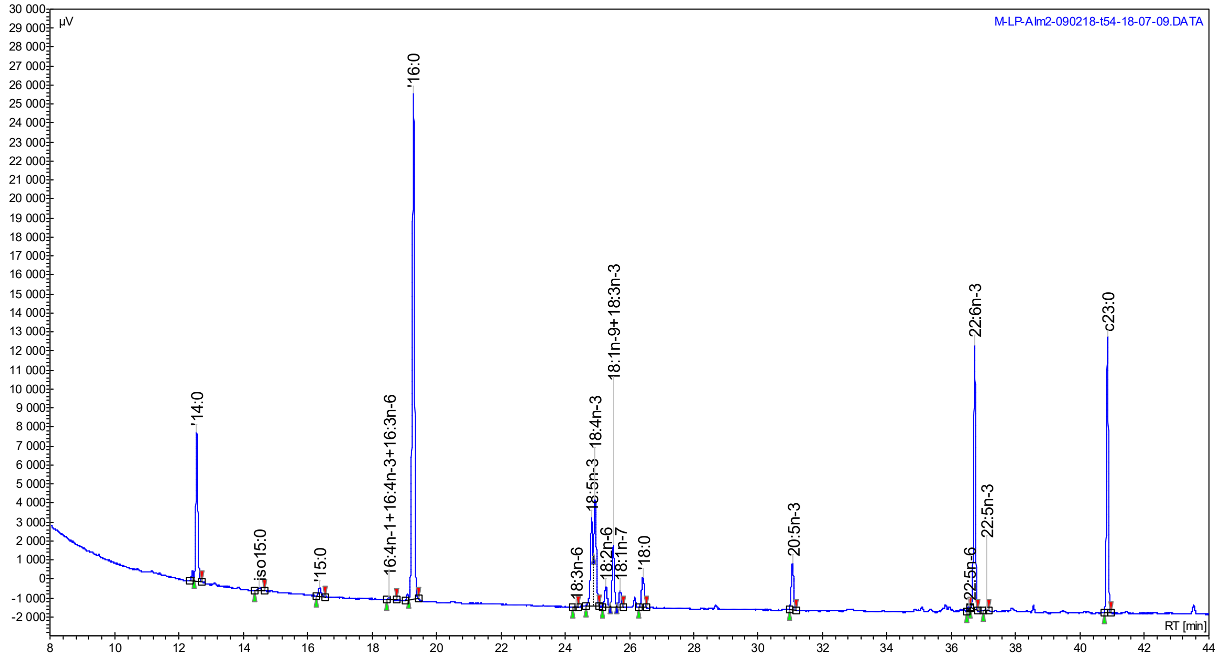

SUPELCO 37 – DB-WAX [A] scale 80000 µV [B] scale 20000 µV

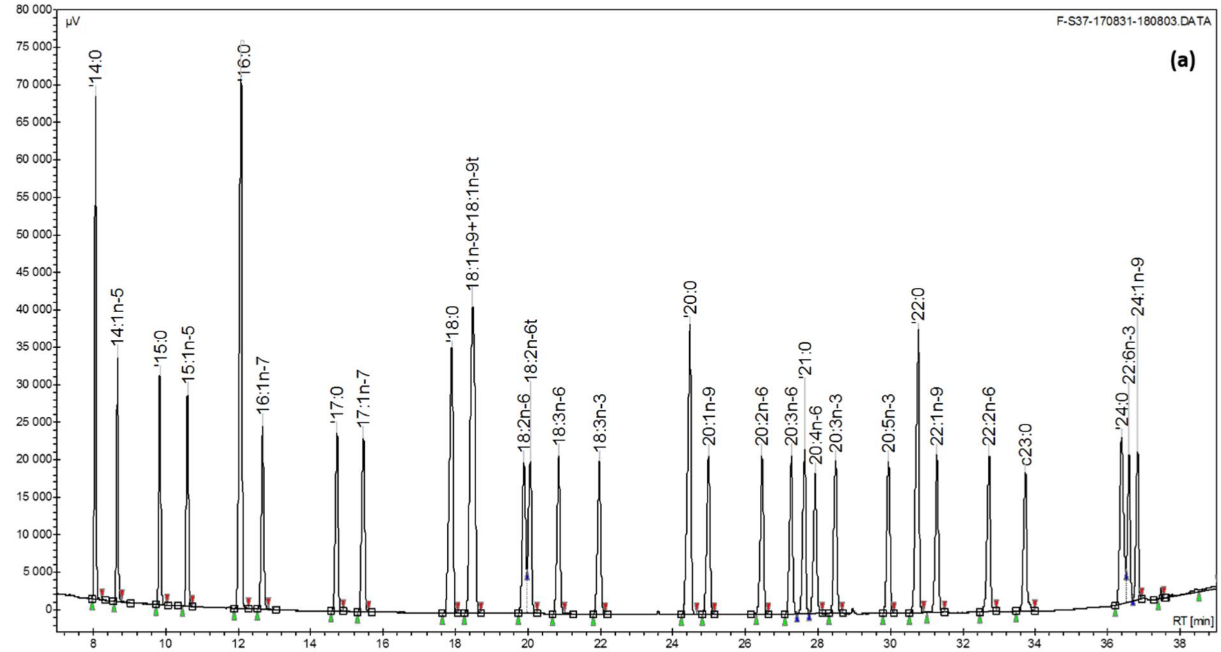

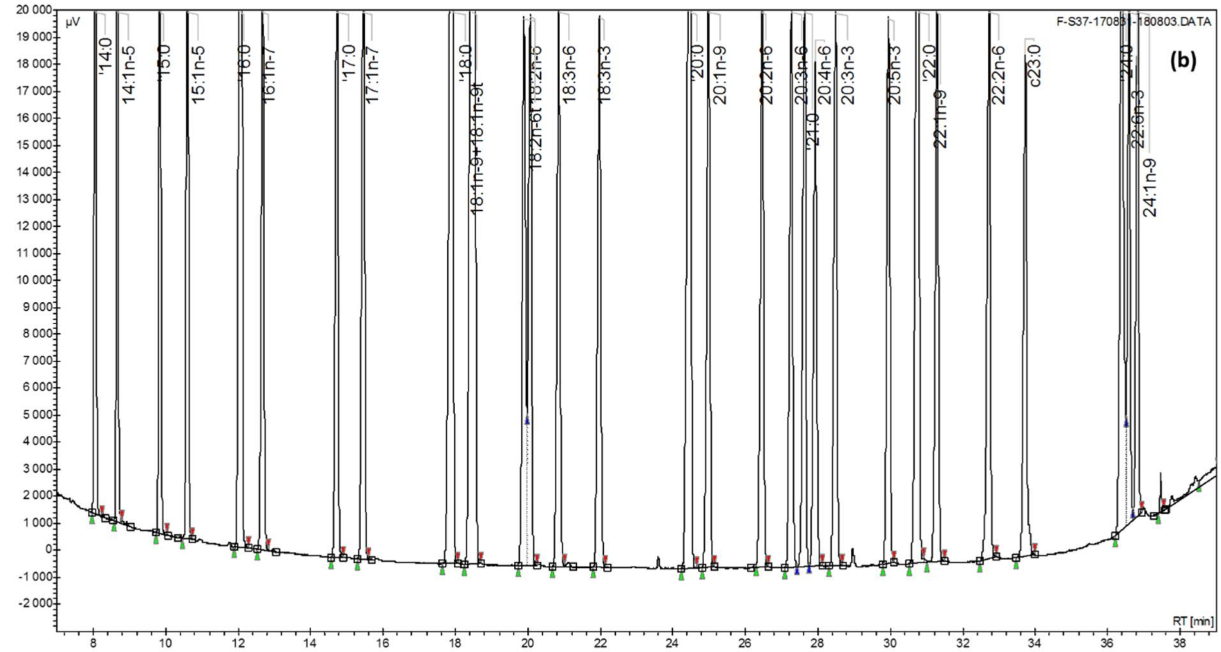

PUFAs No.1 – DB-WAX [A] scale 80000 µV [B] scale 20000 µV

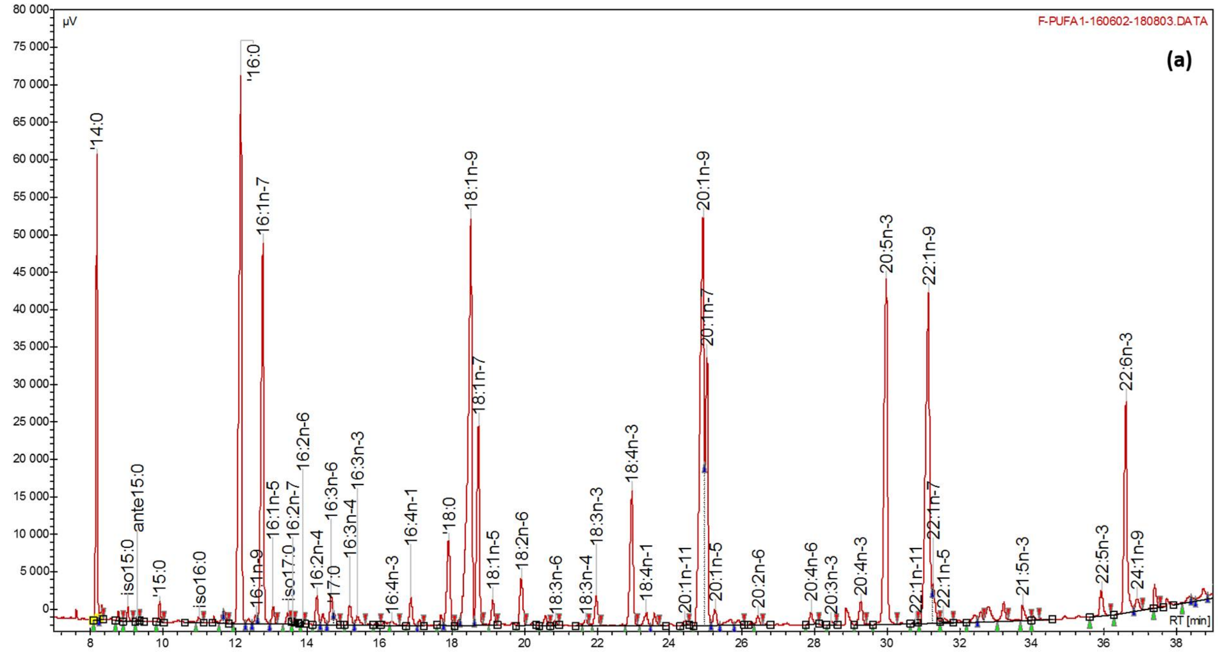

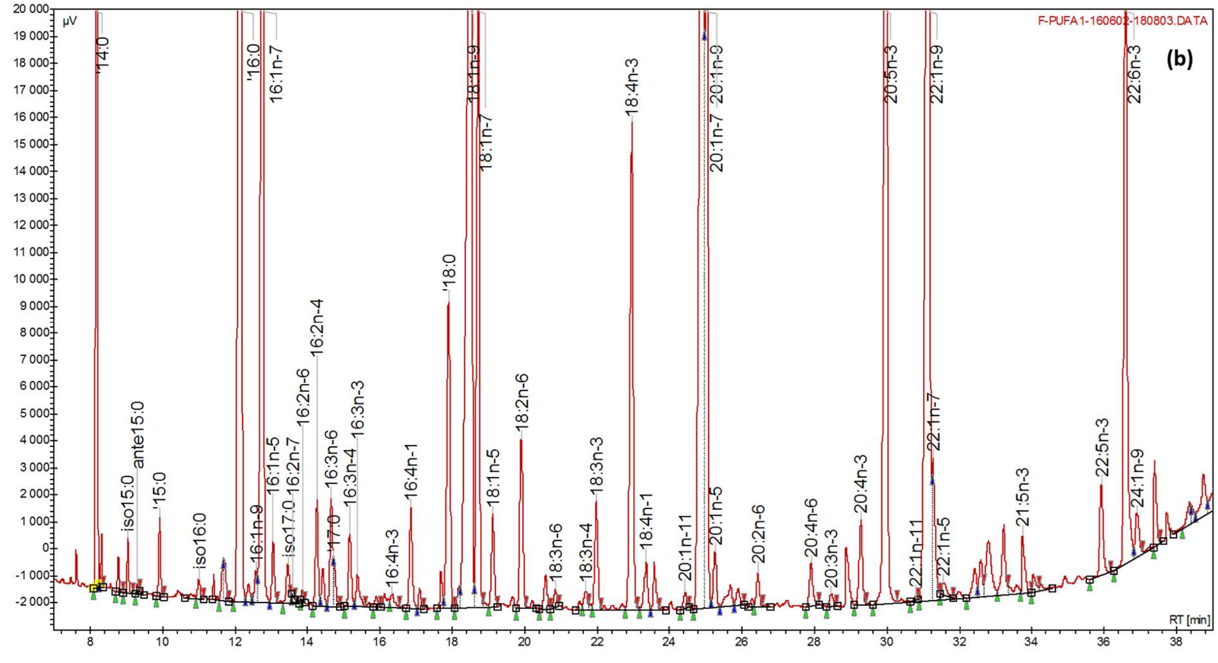

PUFAs No.3 – DB-WAX [A] scale 100000 µV [B] scale 20000 µV

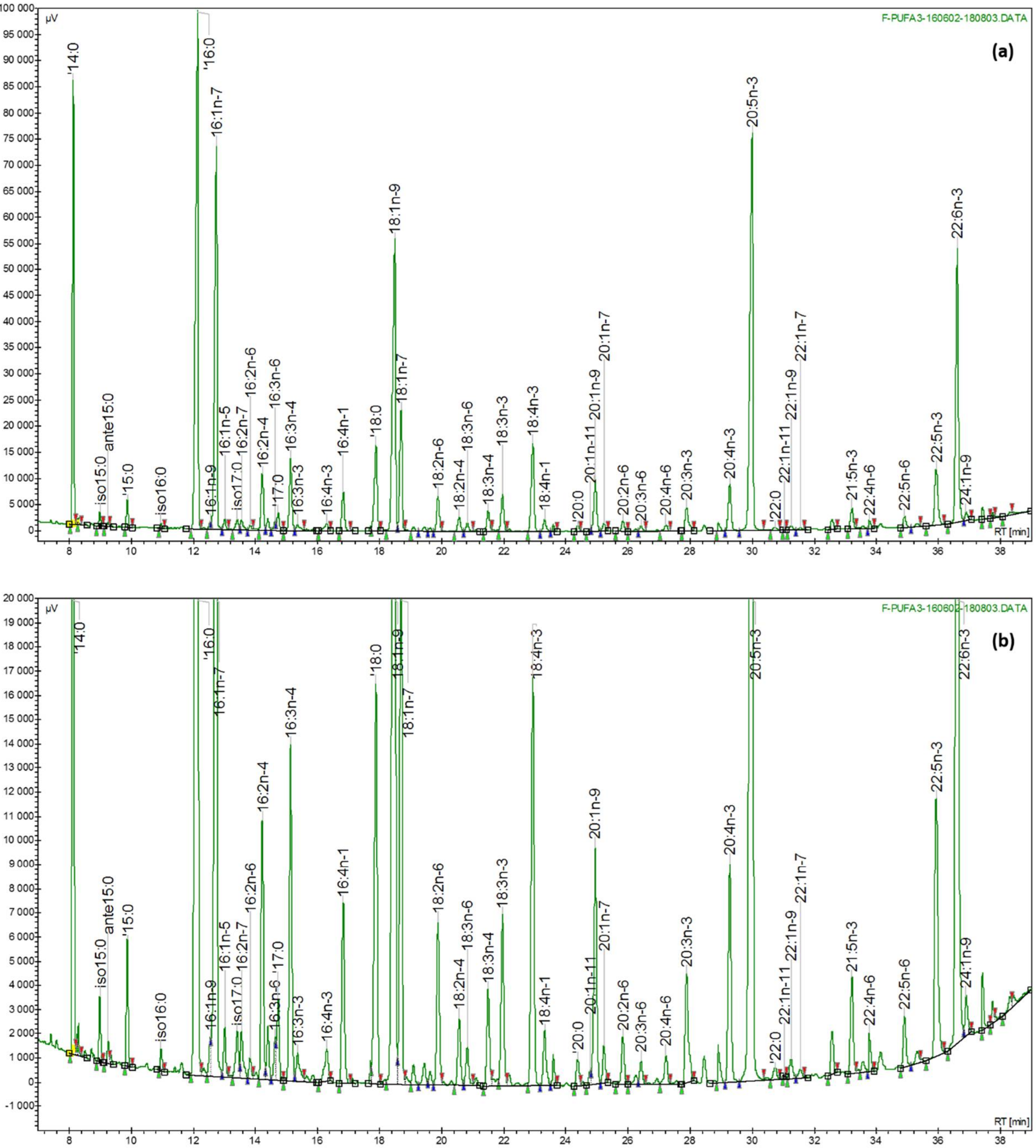

Lab standard (oyster spermatozoa)– DB-WAX (18.12.2018) scale 20000 µV

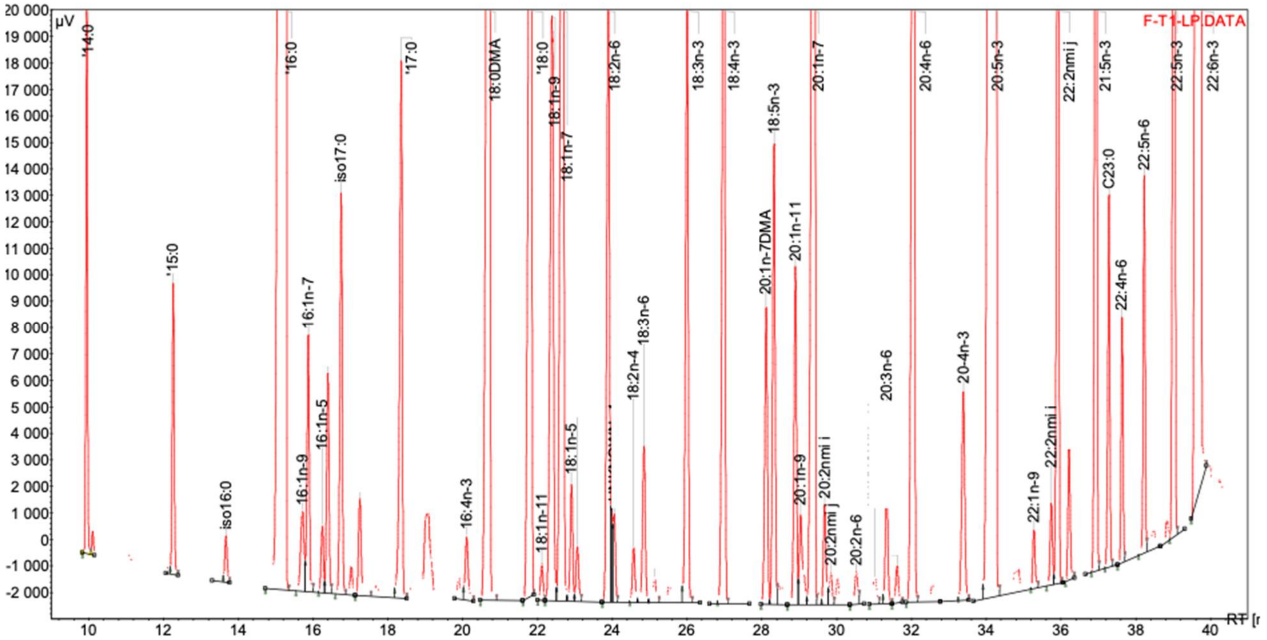

Lab standard (microalgae heterocapsa) – DB-WAX (11.06.2019) scale 20000 µV

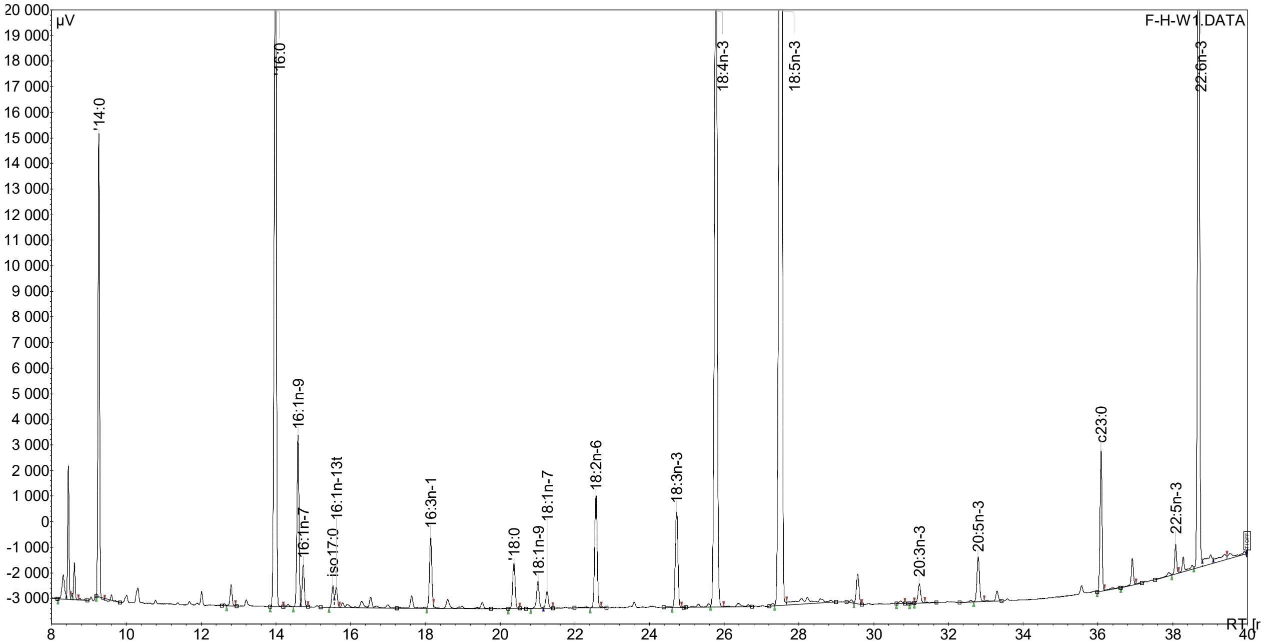

SAMPLE *Alexandrium minutum* Polar Lipid (PL) – DB-WAX scale 20000 µV

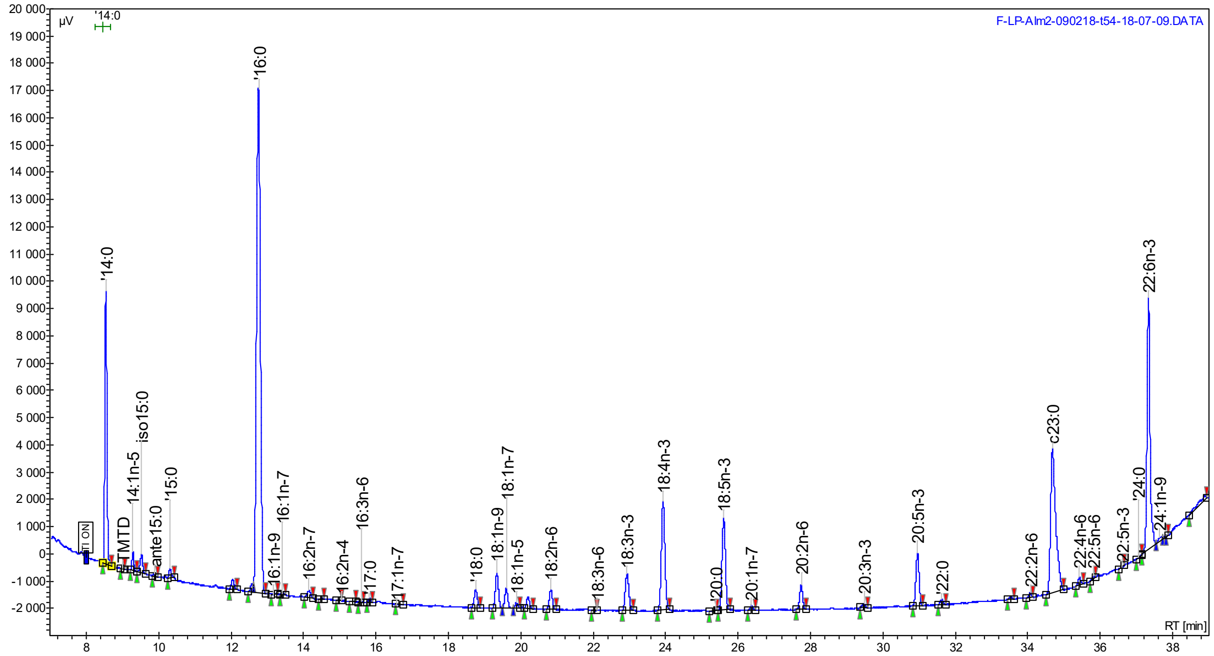

SAMPLE *Alexandrium minutum* Neutral Lipid (NL) – DB-WAX scale 10000 µV

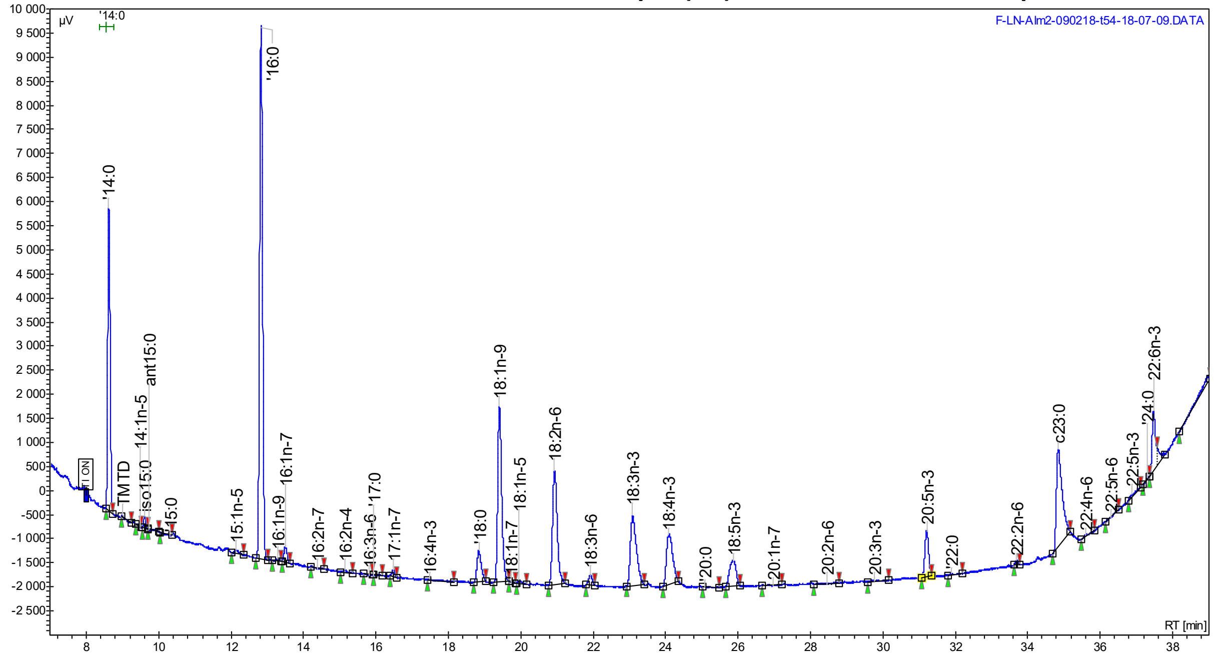

SUPPLEMENTARY DATA – EXAMPLES OF GC-c-IRMS CHROMATOGRAMS (focus on 18:4n-3 and 18:5n-3)

Below are examples made with GC-c-IRMS fatty acid profiles of ^{13}C enriched culture of *A. minutum* at t_0 (Alm1 t_0 sample, with no ^{13}C incorporation) and at 48h (Alm1 t_{48} PL sample). The first graph is the full chromatogram as it was produced after GC-c-IRMS analysis, the second is a zoom made over the C18 FA region of the chromatogram at two different Y scales for the ^{13}C enriched samples.

In Alm1 t_0 PL chromatogram, it corresponds to "natural" isotope (^{13}C enrichment has not started yet) composition of CO_2 with 44, 45, 46 with similar peak intensities because their signals are respectively amplified by 1, 100 and 300 (classical setting in IRMS analysis). Looking at the Alm1 t_{48} PL sample, the 46 is becoming predominant (see chromatogram with the 45000 scale) attesting of ^{13}C incorporation in the FA of the microalgae. In the chromatogram with the 2000 scale, we can see that 44 and 46 masses of CO_2 are in the same ranges as in the Alm1 t_0 PL sample (with ^{13}C incorporation).

Then, through the processing described in the material and methods, the atomic enrichment (AE) can be quantified in the major FA of both neutral and polar lipids from *A. minutum* culture.

Full chromatogram (Alm1 t_0 PL)

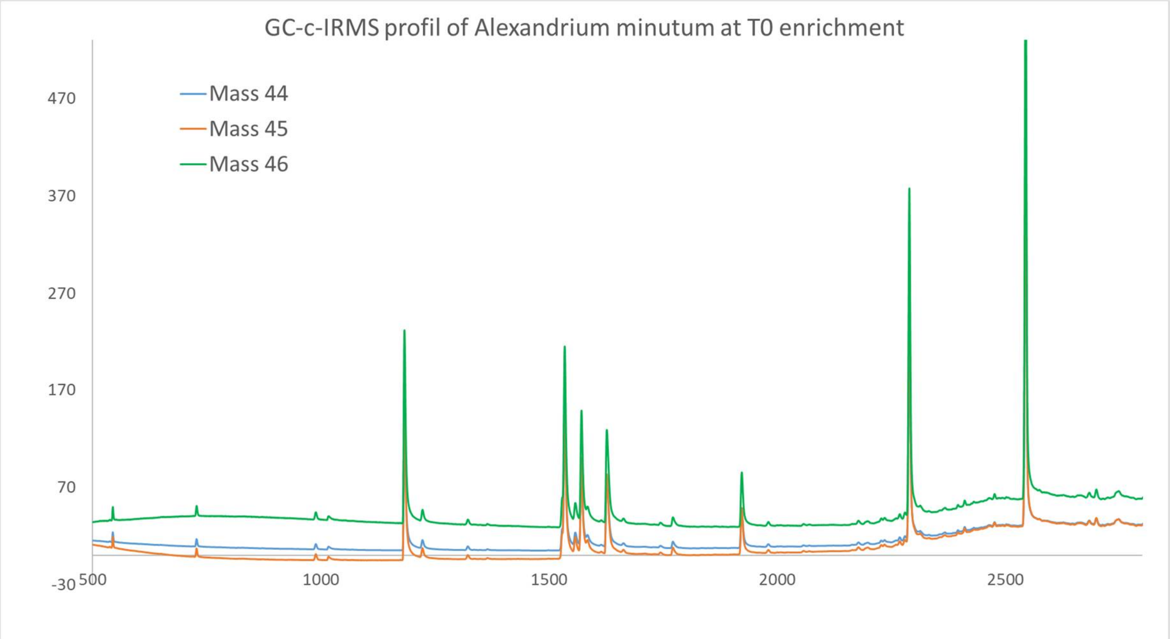

Zoomed chromatogram, C18 FA region (Alm1 t_0 PL)

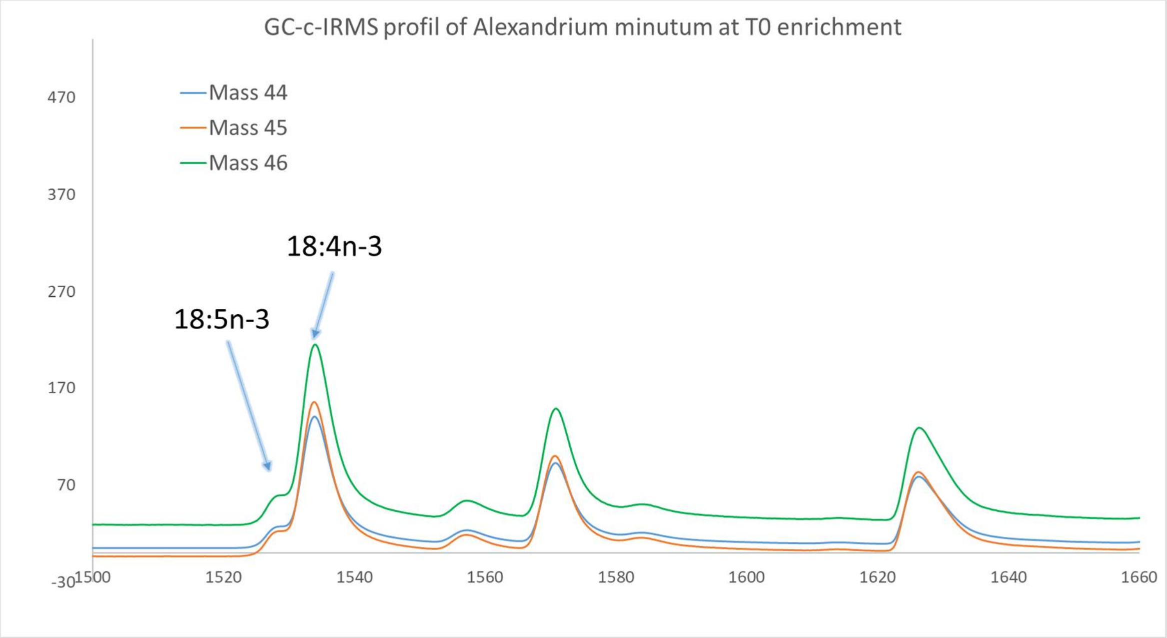

Full chromatogram (Alm1 t_{48} PL)

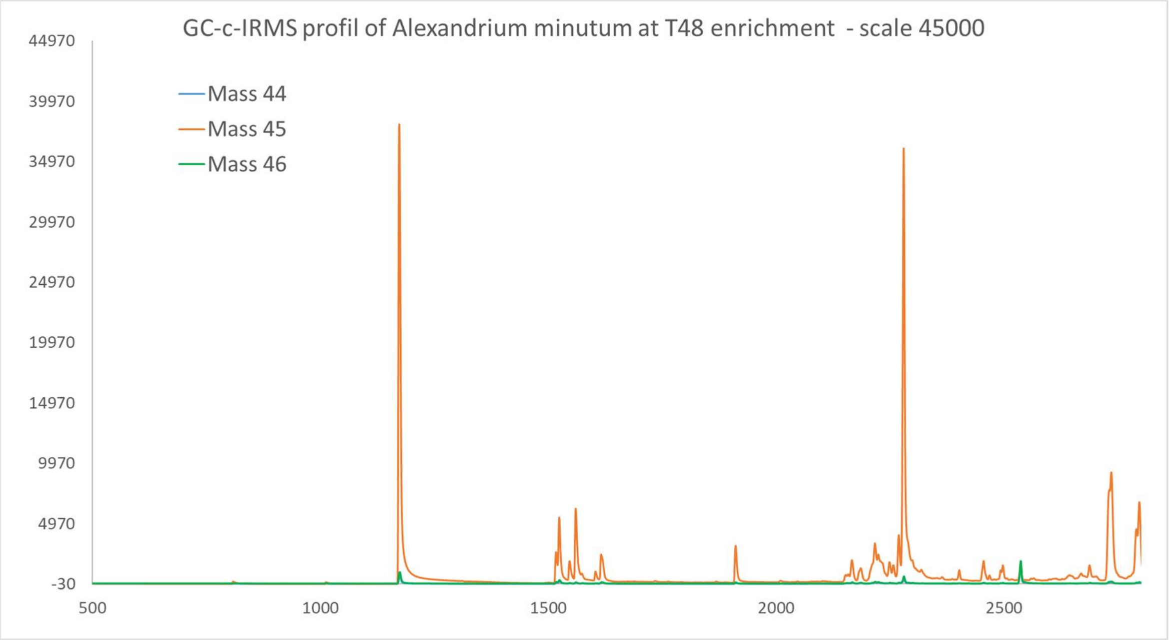

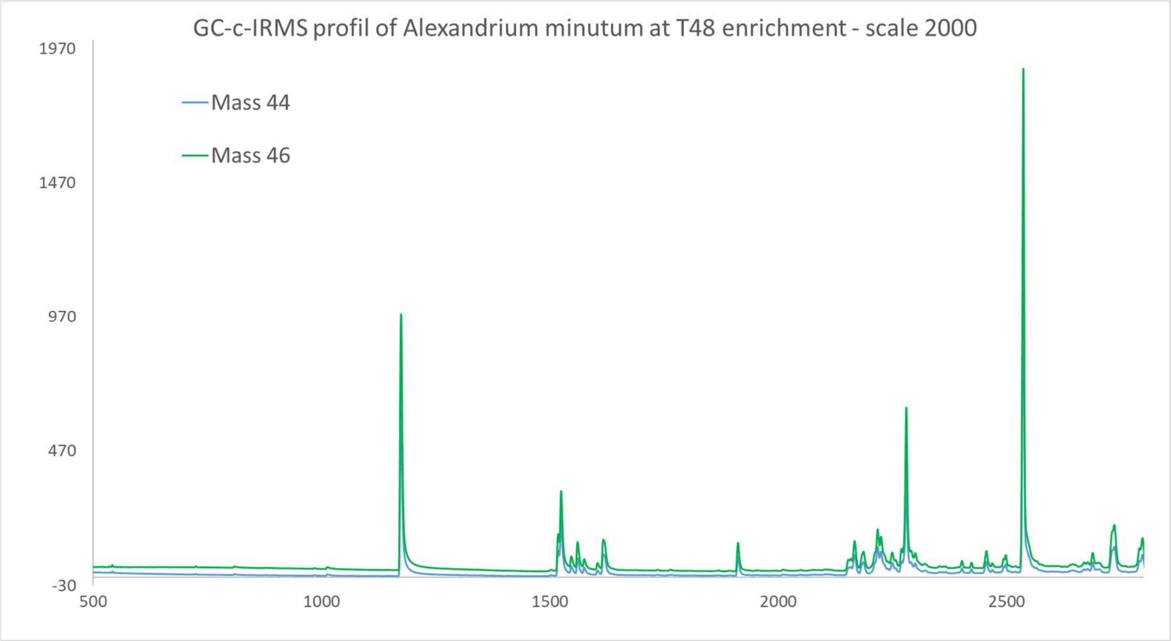

Zoomed chromatogram, C18 FA region (Alm1 t_{48} PL)

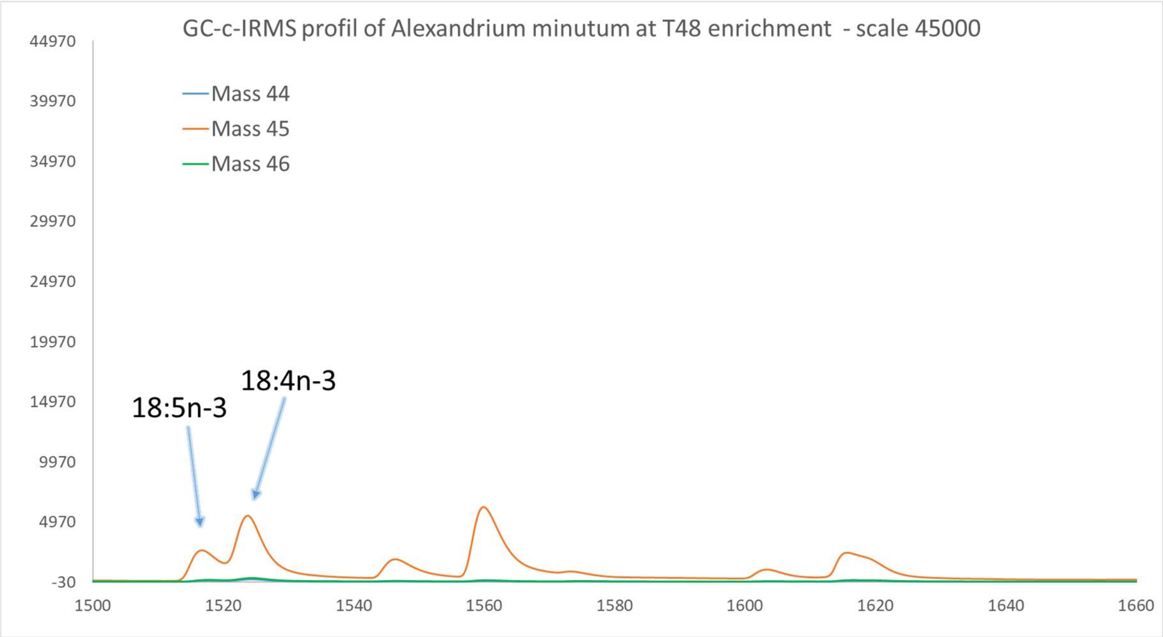

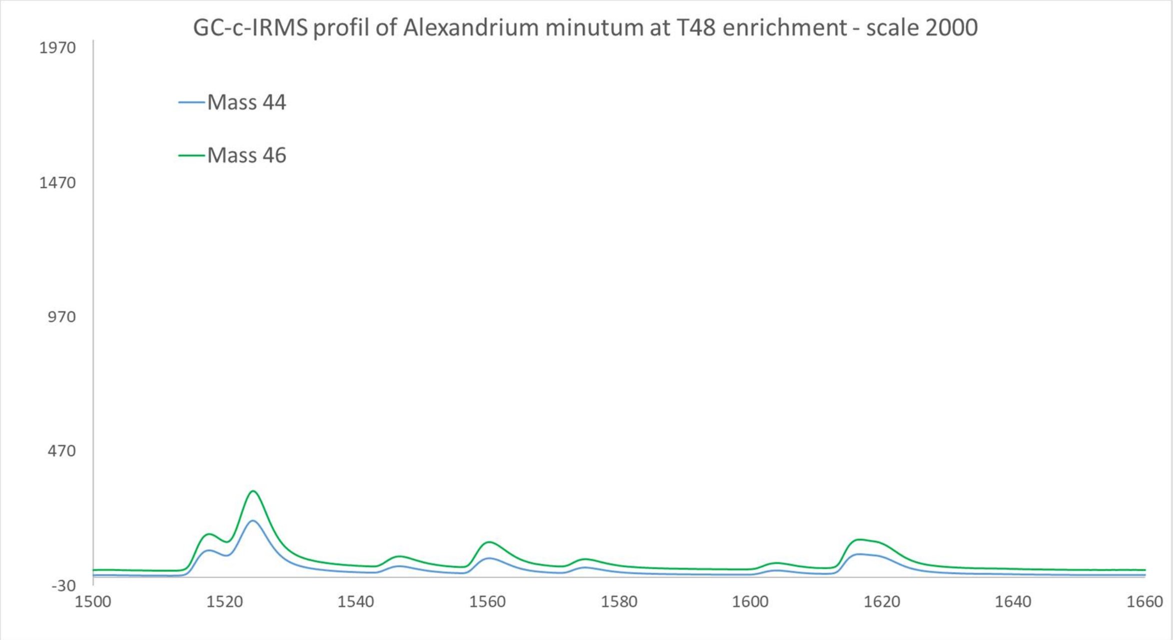

We confirmed identification of 18:5n-3 using GC-MS. GC-MS was performed on each sample to verify with mass spectra the correct identification of the FA. Below are the mass spectra of the 18:4n-3 and 18:5n-3 found in PL samples of *A. minutum*. The following figures compared the mass spectra obtained for our samples on the apolar column for 2 fatty acids. For each, we also attached the mass spectra of FAME available in the LipidWeb website (available in: https://www.lipidhome.co.uk, Christie, 2020).

Full chromatogram for *A. minutum* (PL at t_{54}). Grey marker localizes the 18:4n-3 & 18:5n-3 peaks.

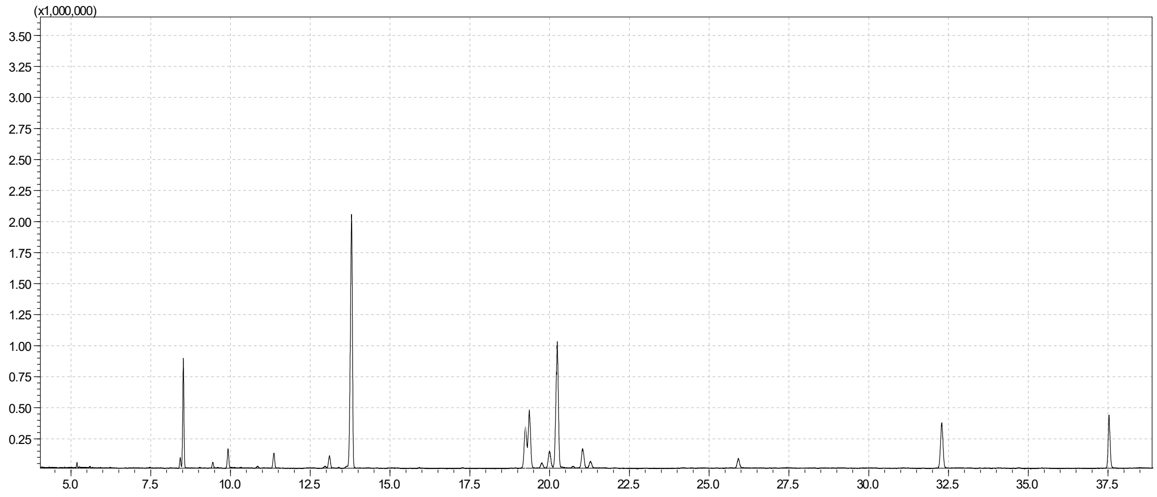

**Peak IDs & RT**		
	RT 8.5: 14:0	RT 20.3: 18:1n-9+18:3n-3
	RT 10.0: 15:0	RT 21.1: 18:0
	RT 13.8: 16:0	RT 25.9: 20:5n-3
	RT 19.2 : 18:5n-3	RT 32.3: 22:6n-3
	RT 19.4 : 18:4n-3	RT 37.5: C23:0
	RT 19.8: 18:2n-6	

Below zoom chromatogram over the zone of interest (C_{18} FA). **Grey marker over 18:5n-3 peak**.

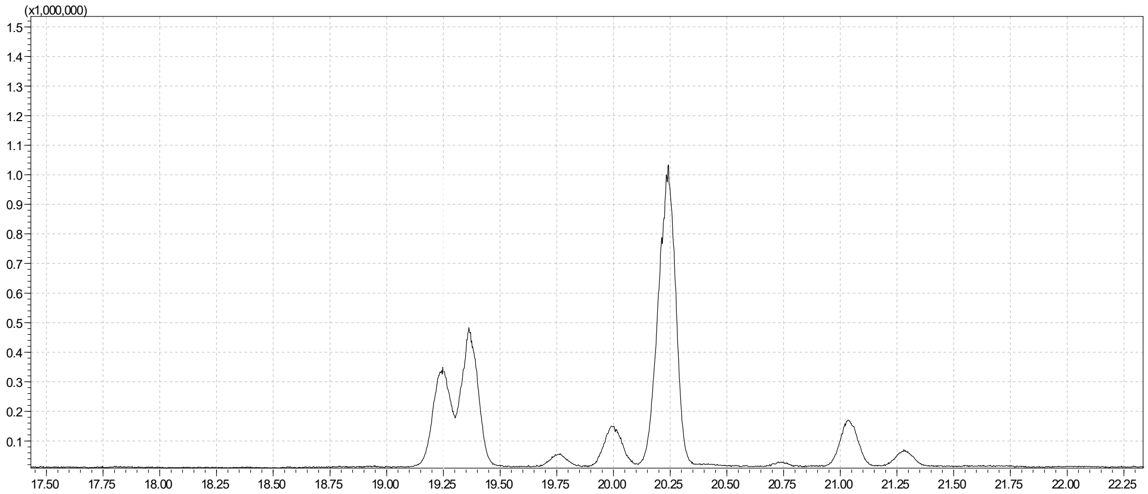

Mass spectra **18:5n-3**

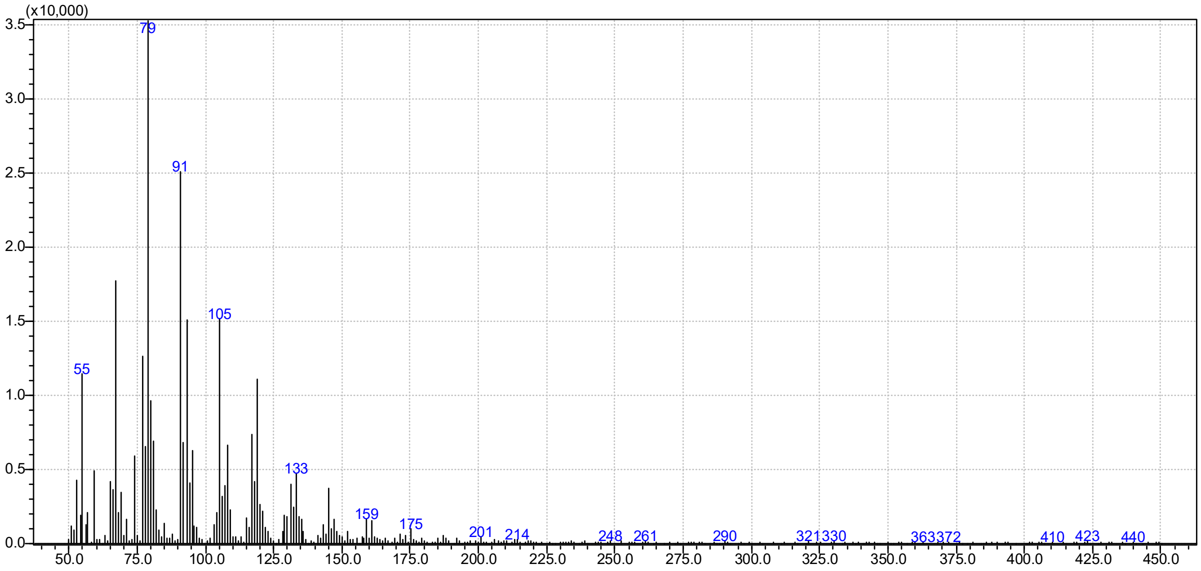

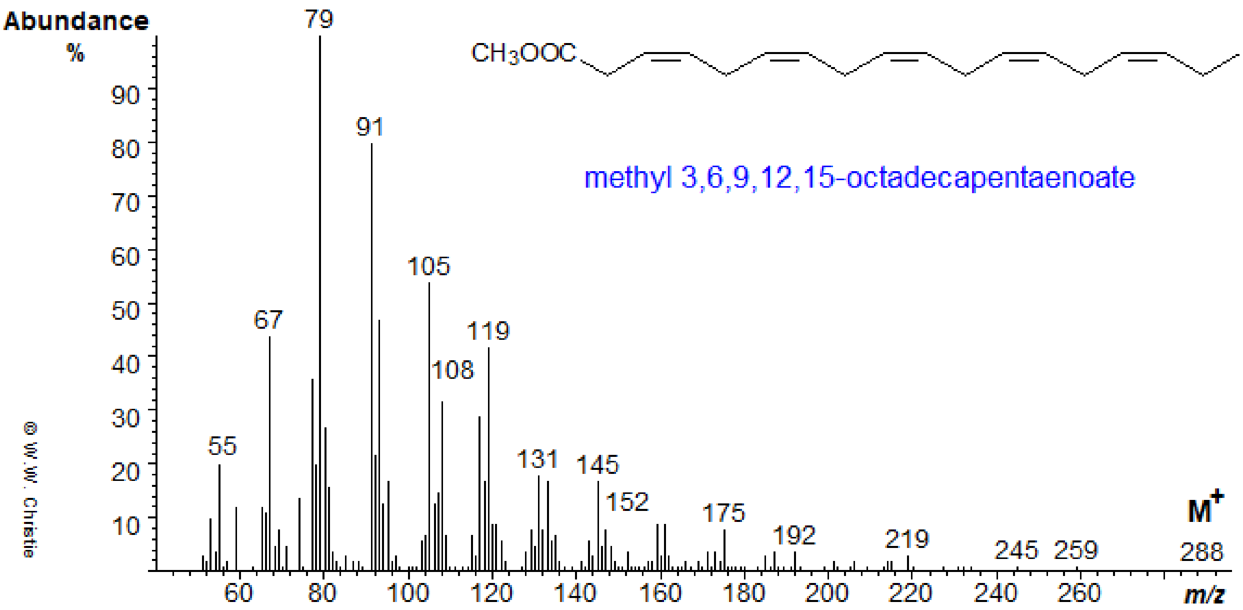

Mass spectrum of methyl octadec-3,6,9,12,15-pentaenoate (18:5 (*n*-3))

The LipidWeb - LipidMaps®

Source: marine algae - donated by Prof. Otto Grahl-Nielsen

Below zoom chromatogram over the zone of interest (C_{18} FA). **Grey marker over 18:4n-3 peak**

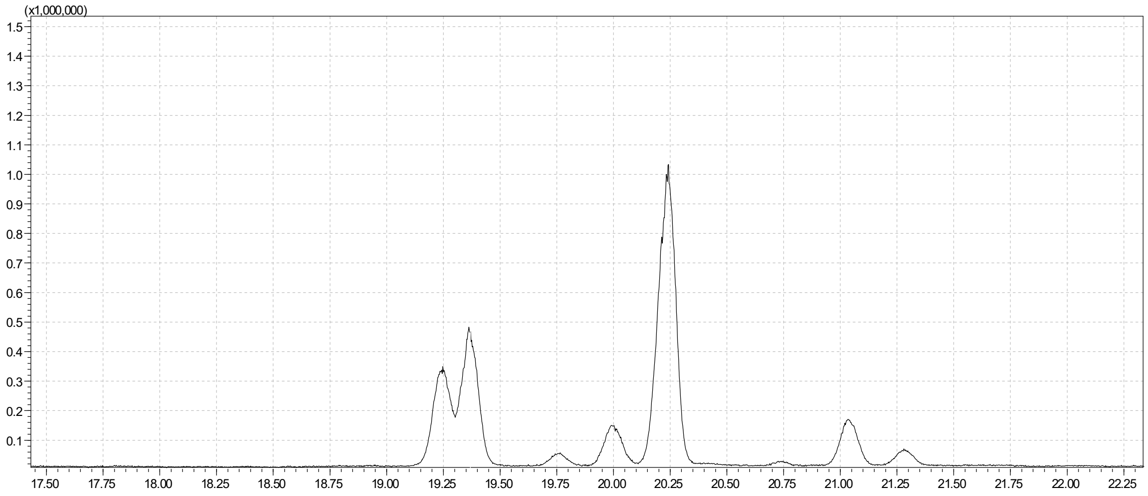

Mass spectra 18:4n-3

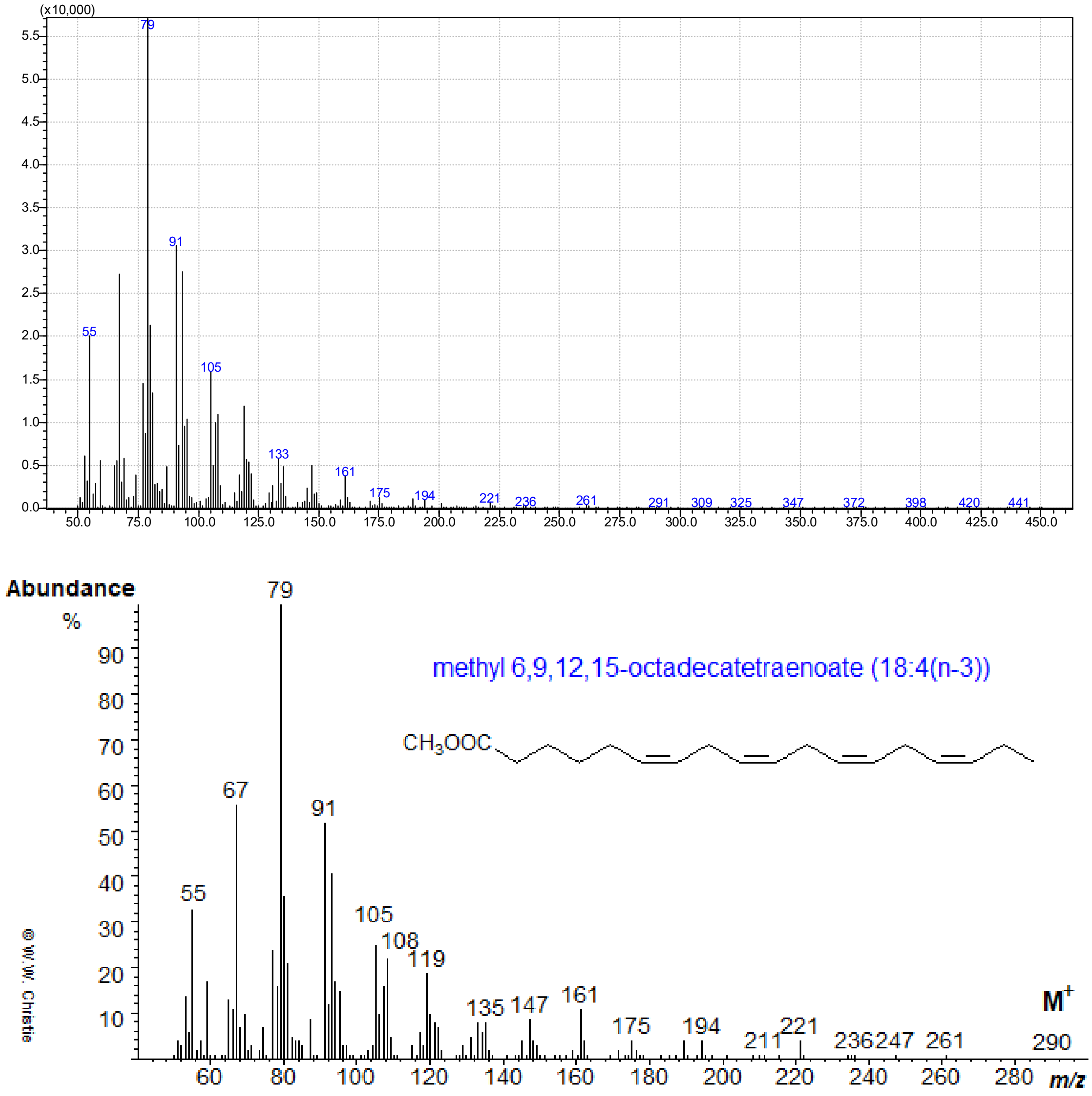

Mass spectrum of methyl octadec-6,9,12,15-tetraenoate
(18:4 (*n*-3) or stearidonate)

Source: red currant oil

The LipidWeb
- LipidMaps®
